# Effect of Cocoa and Cocoa Products on Cognitive Performance in Young Adults

**DOI:** 10.3390/nu12123691

**Published:** 2020-11-30

**Authors:** María Angeles Martín, Luis Goya, Sonia de Pascual-Teresa

**Affiliations:** Departamento de Metabolismo y Nutrición, Instituto de Ciencia y Tecnología de Alimentos y Nutrición (ICTAN-CSIC), C/José Antonio Nováis, 10, 28040 Madrid, Spain; amartina@ictan.csic.es (M.A.M.); luisgoya@ictan.csic.es (L.G.)

**Keywords:** flavanol, cocoa, cognition, cardiovascular, polyphenol, literature review

## Abstract

Increasing evidence support a beneficial role of cocoa and cocoa products on human cognition, particularly in aging populations and patients at risk. However, thorough reviews on the efficacy of cocoa on brain processes in young adults do not exist precisely due to the limited number of studies in the matter. Thus, the aim of this study was to summarize the findings on the acute and chronic effects of cocoa administration on cognitive functions and brain health in young adults. Web of Science and PubMed databases were used to search for relevant trials. Human randomized controlled studies were selected according to PRISMA guidelines. Eleven intervention studies that involved a total of 366 participants investigating the role of cocoa on cognitive performance in children and young adults (average age ≤25 years old) were finally selected. Findings from individual studies confirm that acute and chronic cocoa intake have a positive effect on several cognitive outcomes. After acute consumption, these beneficial effects seem to be accompanied with an increase in cerebral blood flow or cerebral blood oxygenation. After chronic intake of cocoa flavanols in young adults, a better cognitive performance was found together with increased levels of neurotrophins. This systematic review further supports the beneficial effect of cocoa flavanols on cognitive function and neuroplasticity and indicates that such benefits are possible in early adulthood.

## 1. Introduction

A healthy lifestyle is essential for the maintenance of normal cognitive functions both in young and elderly populations [1,2]. Cognitive abilities, particularly memory, attention, execution, and processing speed are progressively declined throughout the adult lifespan [3]. A complex interplay of genetic, endogenous, and environmental factors determine this process, with aging being the greatest risk factor for cognitive deterioration and dementia [4]. Increasing data suggest that lifestyle approaches such as nutrition may represent a promising opportunity to slow or prevent the progressive cognitive decline [5,6]. Indeed, there are numerous evidences that certain dietary patterns, as well as physical activity, can improve cognitive abilities and brain health across the human lifespan [7,8,9]. In this context, the role of plant-based dietary patterns and polyphenol-rich plant foods on either preventing or improving cognitive function has become an emerging area of investigation [10].

Considering this, recent reports have indicated that polyphenols could be associated with beneficial effects on cognitive and brain functions in young [11] and older adults [12]. Consistent findings from several studies confirm that these natural compounds can induce a positive effect on several cognitive processes, specifically executive function, attention, working memory, and processing speed [13]. Moreover, polyphenols can also exert neuroprotective functions due to their effectiveness against oxidative stress and inflammation as well as their capacity to modulate prosurvival or antiapoptotic signaling pathways [14]. Interestingly, better beneficial effects of polyphenols on brain plasticity biomarkers and on different cognitive functions have been found in young and middle-aged adults than in elderly [12,15], suggesting that young people can be an attractive population target to prevent onset of age-related diseases and cognitive decline. Indeed, cognitive function in young adults has great potential for intervention since brain tissue is less damaged and shows greater plasticity in response to new challenges. 

In this context, cocoa is considered one of the best-known sources of dietary polyphenols, mainly monomeric flavanols such as (−)-epicatechin and (+)-catechin, as well as their dimers procyanidins B2 and B1 [16]. Furthermore, in recent years, evidences from human clinical studies have suggested that cocoa and cocoa-derived products consumption can be effective to improve general cognition and working memory, particularly among older population at risk or with cognitive decline [17,18]. Indeed, in a very recent systematic review [19], it has been indicated that memory and executive function can increase significantly after intake of intermediate doses of cocoa flavanols (CF) (500–750 mg/day). All these findings provide strong evidence supporting a potential beneficial role of cocoa to reduce cognitive decline and sustain cognitive abilities. However, to date, the majority of studies have focused on the cognitive effects of cocoa flavanols in older people, while studies investigating the efficacy of cocoa on brain processes in young adults are more limited. Because young people have been identified as a particularly attractive population for cognitive interventions [12], it would be necessary to further investigate the impact of cocoa intake in young healthy adults as a potential preventative strategy to prevent cognitive decline related with age.

Therefore, the main objective of this study was to revise and update results from recent studies reporting beneficial effects of cocoa flavanols on brain function and cognitive response that might confirm in young humans most of the findings previously observed in adults and aged people. To this end, this systematic review has focused on clinical trials investigating the effect of acute (cognition assessment is taken less than 4 h after cocoa flavanols intake) and chronic (cocoa flavanols are consumed at least for 5 days and cognition assessment is taken at least after overnight refrain from cocoa or other flavanol-rich foods consumption) cocoa intake on cognition in young adults (average age ≤25 years). In this study, methodological aspects as well as the impact of cocoa intake on cardiovascular and cognitive endpoints are discussed. This review provides further support to the effect of cocoa on cognitive function and suggest for the first time that such benefits can be also observed in younger people. 

## 2. Materials and Methods 

### Literature Selection

The bibliographical search was conducted in August 2020 in the Web of science. The search was taken with the following terms: “neuronal OR cognition OR memory OR learning OR psychological” AND “cocoa OR chocolate”. In this case a total of 654 articles were found, of which 518 were research articles and the rest were reviews, book chapters, etc. In every case, the search of the specified terms was done under the search field “topic”. When limiting the search by including to the previous terms: AND “adolescent OR young OR children OR youth” the number of items was dramatically decreased to 96 of which 83 were research articles. When performing the same search but in PubMed we got initially 718 results (of which 89 were randomized controlled trials) and when including in the search the terms “adolescent OR young OR children OR youth” we got 268 of which 61 were randomized controlled trials.

**Figure 1:**
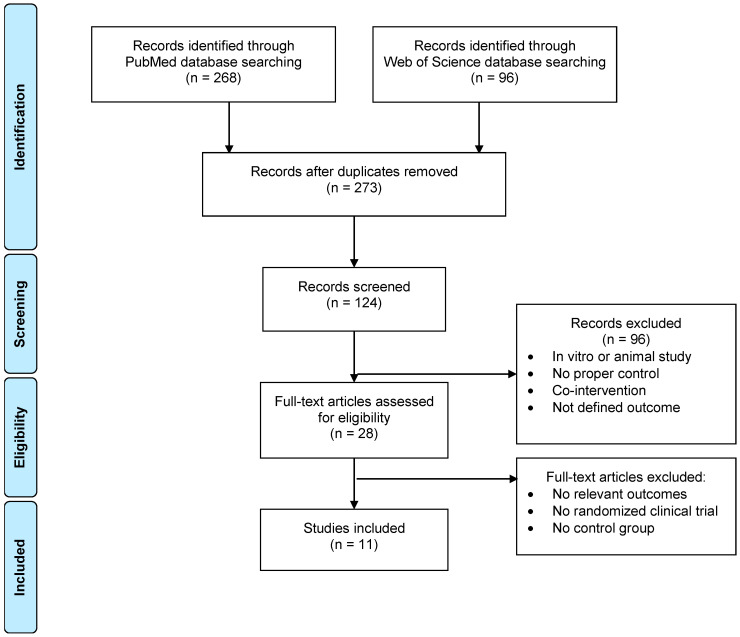
PRISMA 2009 Flow Diagram [20] for the review on the effect of cocoa and cocoa products on cognitive performance in children and young adults.

After merging the two data lists from Web of Science and PubMed and reviewing only the title of the papers we ended up with 124 papers (Figure 1). Most studies were eliminated because already in the title they showed no relation with our search. In many cases, chocolate was used as an appealing food in animal and human studies, other were cell based studies and in a few “cocoa” was the acronym of the study (with no relation with our interest) or the name of one of the authors. In the next step, we double checked the abstracts of the papers to end up with only 28 papers that were read in full. Most of the papers from the 124 initially selected were eliminated because chocolate was used as an appealing food or image for go-no-go assays in both animals and/or humans. In others, chocolate was included together with suits or soft drinks as unhealthy foods in epidemiological studies. Finally, we only selected 11 of these papers that are summarized in Table 1, and all of them are intervention studies in which children or young adults averaging no more than 25 years ingested cocoa or chocolate. The decision of including studies up to 25 years of average age was due to the fact that only in one study children averaging 10 years old were included, the rest were done in young adults averaging the mentioned age. From the 28 papers read in full, most were eliminated because they did not include any control or placebo or because in the description of the cocoa or chocolate used in the study there was no mention of the polyphenol or flavanol content.

## 3. Results and Discussion

The literature initially selected on chocolate and cocoa effects on cognition and neurological endpoints allowed us a view of the bigger picture, including animal studies and human trials conducted in adults and aged population groups. As detailed below, one of the mechanisms responsible for the beneficial effects of regular cocoa intake seems to be the ability of flavonoids to modulate signaling pathways promoting neuronal function and brain connectivity [11,21]. In addition, cocoa also may improve cerebral blood flow (CBF), inducing changes in processing of memory [18]. A third mechanism of action has now emerged through the gut–brain axis and the ability of gut microbiota to improve the bioavailability of polyphenols [22]. Colonic bacteria catabolize undigested polyphenols to produce more active and better-absorbed metabolites, with possible direct neuroprotective potential. Indeed, data from animal studies have shown that epicatechin and various flavanol microbial metabolites [23,24,25] as well as theobromine [26] cross the blood brain barrier, suggesting that they could act directly on the brain to lead to cognitive improvements. A few other potential direct and indirect mechanisms have been proposed to explain the beneficial effects of phenolic compounds on brain function (for a review see [27]).

Cognitive function, defined as the mental performance that enables information processing, applying knowledge, and changing preferences [28], has been evaluated in animal models after cocoa polyphenols intake and isolated epicatechin, the main phenolic component of cocoa, through standardized behavioral tests. Results of isolated epicatechin intake in animal models have evidenced improvements in spatial memory, angiogenesis, and neuronal spine density [29]. An increase in the expression of genes previously associated with learning and a decrease of markers of neurodegeneration have also been observed in rodents after polyphenols intake [29].

These animal models have contributed to characterize some mechanisms triggered by cocoa polyphenols in specific tissues [30,31] and confirmed that monomeric CF and their microbial metabolites cross the blood–brain barrier and have been localized in the brain, particularly in areas connected to learning and memory, such as hippocampus, cerebral cortex, cerebellum, and striatum [31,32]. Epicatechin is readily absorbed by the human digestive system reaching blood plasma concentrations peaking at 2–3 h after consumption; a significant proportion of circulating epicatechin levels could cross the blood–brain barrier and have the capacity to act directly on the brain, which could potentially lead to cognitive enhancement [29].

Having all the previous information in mind and now focusing in the eleven papers that were selected and that included only studies taken in children and young adults, we will discuss different aspects related to the methodology used, the cardiovascular implications, and the neurocognitive effects of cocoa.

### 3.1. Methodological Aspects

The maximum average age for the inclusion of studies in this review was finally fixed to an average of 25 years. It should be noted, in this sense, that from the eleven studies selected only one included children (see Table 1). The eleven intervention studies involved a total of 366 participants. All but two included males and females in the sample studied in different proportions from 30% to 73%. In the study by Francis et al. (2006) [33], only women were included, and they found a positive effect of cocoa polyphenols on blood oxygenation in active brain regions by means of functional magnetic resonance imaging (fMRI). In that of Tsukamoto et al. (2018) [34], only men were included, and the main result was an enhancement of exercise-induced improvement in executive function. On the other hand, the only study in which the statistical treatment of the data had taken into consideration the effect of sex on different parameters related to the cognitive functions in sleep deprivation conditions showed a beneficial effect of CF on working memory accuracy only in women [35].

Out of the eleven studies that were finally selected for this thorough review on the effect of cocoa and cocoa products on cognitive performance in young adults, only four can be considered chronic studies. From these four, only that of Sumiyoshi et al. (2019) [36] can be considered strictly chronic (30 days), because it is the only one in which the design included a period of 20 h in which no chocolate, cocoa, or other source of polyphenols was consumed. In the other three studies, even if the cocoa or chocolate source of polyphenols was consumed for 5 days in the case of Francis et al. (2006) [33], for 9 to 24 days in the case of Calderon-Garciduenas et al. (2013) [37], and for 28 days in that of Massee et al. (2015) [38], in all three the endpoints were measured between 1.5 and 4 h after the last ingestion of the CF source.

Regarding the amount of chocolate or cocoa consumed by participants in the different studies, it ranges from 35 mg of epicatechin contained in 24 gr 70% cocoa chocolate [36] or 85 mg total flavanols contained in 35 gr 70% cocoa chocolate [43] to almost 1 gr CF [39]. 

Most of the included studies refer the effect of CF on different cognitive functions in standard conditions, but other studies use some type of stressor in order to show a positive effect of cocoa. For instance, Tsukamoto et al. (2018) used extreme physical activity [34], Grassi et al. (2016) sleep deprivation [35], Calderon-Garciduenas et al. (2013) pollution [37], and Massee et al. (2015) mental fatigue [38]. This fact might influence the final effect found and the lack of effect in studies where resting condition was used. Additionally, some of the works describe a lack of positive results on cognition outcomes that could be due to the ceiling effect that may be introduced in the assays due to either the excessive cognitive performance of the selected population at baseline or by the effect of training on the cognition task before the starting point of the intervention [33].

### 3.2. Results on Cardiovascular Endpoints

Cardiovascular health has been closely linked to cognitive performance [44], and a number of studies have suggested changes in cerebrovascular function and flow mediated dilation as the main mechanism responsible for the beneficial effect of cocoa phenolics on cognitive function [18].After extensive review of the literature, some authors have concluded that enhanced cognition due to polyphenol consumption is widely caused by the signaling molecule nitric oxide (NO) through two main NO-derived effects: vasodilation and neurotransmission [42,45]. Thus, cocoa flavonoids may facilitate production of NO, which improves vascular endothelial function by relaxing the smooth muscle tissue of blood vessels [46,47,48].

Already in the past decade, some studies showed that CF, especially epicatechin, act directly on the endothelium of brain vessels, stimulating activity of the endothelial nitric oxide synthase (NOS) and generating NO that in turn induces vasodilation and improves cerebrovascular perfusion [49,50,51,52]. Indeed, by stimulating the guanylate cyclase, NO mediates vasodilation in blood vessels including cerebral arteries that results in increased CBF [53]. A study has recently confirmed this same effect for some epicatechin microbial metabolites [54]. Other clinical investigations also revealed that supplementation with flavanol-rich cocoa reliably improves an assortment of cardiovascular variables, such as peripheral blood pressure in prehypertensive and hypertensive patients [55,56,57], as well as in normotensive populations [58]. Moreover, cocoa supplementation increases CBF [50,51] and flow-mediated dilation [59,60,61,62]. In the same line, other authors showed that flavanol-rich cocoa may increase vasodilation in a NO-dependent way resulting in enhanced CBF and blood perfusion throughout the central and peripheral nervous system [51,63,64,65,66,67]; this would afford better supply of oxygen and glucose to the neurons and removal of waste metabolites in the brain and sensory systems [68,69]. Consistent findings from several individual studies confirm that enhanced cognitive performance in healthy young adults is accompanied by an increase in CBF or cerebral blood oxygenation following the consumption of high flavanol cocoa drink [33,70]. For example, 5 days of CF (150 mg/day) supplementation to healthy young females increases the blood oxygenation level-dependent signal intensity in response to a cognitive task and the CBF to gray matter [33]. These effects have been supported by changes in flow-mediated dilation [71] and changes in the concentration of oxygenated blood in the cortex, as measured with near-infrared spectroscopy in the immediate hours after consumption [41].

In the last ten years, other chronic trials ranging from two to three months have shown that daily CF consumption shows an association with positive effects on executive function and working memory in healthy adults [72,73,74]. These chronic effects are shown in parallel with changes in CBF indicating a cerebrovascular mechanism of action. An acute cerebrovascular mechanism also has potential to affect cognitive function in the immediate hours following CF consumption [75]. Indeed, a double-blind crossover design in healthy young adults performed by Scholey and colleagues (2010) [39] showed improvements in executive function up to 1 h following consumption of a flavonoid-rich cocoa drink relative to a low flavonoid control. Moreover, dark chocolate bar consumption has also been associated with acute executive function improvements in adults averaging 24.1 years old [38].

Interestingly, improved cognition by CF has also been related to changes in visual function. Thus, improved spatial working memory in addition to benefits for visual contrast sensitivity has been reported two hours after dark chocolate consumption in healthy young adults [40] indicating that vascular benefits may extend to the retina. The highly vascularized retina, particularly the macula with its substantial projection to the visual cortex, may be most susceptible to enhanced blood flow and increased metabolic supply afforded by polyphenol flavanols in dark chocolate [33,68,76].

Finally, a nutritional intervention with high CF intake increased the benefits to cardiovascular function by moderate to intense exercise, suggesting that the combination of CF consumption and aerobic exercise may be beneficial for improving cognitive function [34]. Similar effects have been also recently reported for similar polyphenols such as green tea flavanols [77].

Given the strong links between vascular health and cognitive function [78], it is possible that cocoa will improve cognitive performance indirectly through improvements to CBF and vascular health. In addition, animal models indicate that CF administration stimulates angiogenesis in the hippocampus [79]. Due to the multiple biological effects of flavonoids, it was recently suggested that all of these mechanisms have a role to play and are interrelated [42], and endothelial NO represents a key molecule in this relationship [80].

### 3.3. Results on Cognition Endpoints

Animal studies have shown that the absorbed flavonoids directly interact with a number of cellular and molecular targets in the brain, exerting pronounced antioxidant effects and improving brain tissue and function in the regions mainly implicated in learning, memory, and cognition [81]. This suggests a potential neuro-modulatory and neuro-protective role for CF and their significance for cognitive and affective function, executive control, and behavior. However, only few human studies so far have specifically addressed neurobiological, cognitive, affective, and behavioral effects of flavanol-rich cocoa products. At cell and molecular level, neurobiological impact of flavanols on the brain, learning, memory, and cognition seem to occur in two major ways: first, flavanols can specifically interact within a number of cellular signaling pathways, primarily with mitogen-activated protein (MAPK), extracellular-signal-regulated (ERK), and phosphoinositide 3-kinase (PI3-kinase/Akt) signaling cascades [82,83]. These cascades trigger gene expression and protein synthesis for maintaining long-term potentiation and establishing long-term memories [82]. Flavonoids modulate the transcription factors engaged in signal transduction through protein-kinase inhibition [53,84] and promote the expression of BDNF that is critical for neurogenesis, also in adult animals, synaptic growth and neuron survival, especially in the learning- and memory-related brain regions such as the hippocampus and sub ventricular zone [85,86].

On the other hand, independent of CBF effects through activation of NO synthesis, CF also influences neuronal signaling pathways where NO acts as a neurotransmitter [45]. This effect offers an alternative explanation for the enhanced cognition following polyphenol consumption. Actually, in a recent randomized, double-blind, placebo, and baseline-controlled crossover study, Karabay and colleagues showed that rich polyphenol supplementation enhances the majority but not all of cognitive functions, suggesting that the modulations of cognitive functions in response to polyphenol supplementation are more related to neurotransmission rather than vasodilation [42]. Thus, previous reports indicate that natural flavanol/catechins can be associated with an increased expression of BDNF and higher cognitive function [87] and changes in BDNF have also been observed following twelve weeks of CF consumption [88]. However, Decroix et al. (2016) investigated the effect of 900 mg CF on neuroplasticity but failed to show a significant acute or delayed effect on BDNF [70]. A recent clinical trial supports an increase in BDNF levels related to the effect of different polyphenol supplementation that was accompanied by improved cognitive function [88]. 

## 4. Conclusions

The studies conducted so far on the potential of CF as neuroprotectors and neuromodulators have evidenced a certain efficiency on cognition and behavior, both in acute and in sub chronic (for several weeks) or chronic modes. The immediate effects can be achieved with a single dose of CF in appropriate dosages. The long-term effects most likely require chronic intake of flavanol-rich products. In general, flavanol active doses range from less than 100 mg to around 500 mg.

Most studies support the key role of NO and its effect on endothelial tissue to improve brain blood flow to increase cognitive function and attention. However, NO biological role in cognition is not solely as vasodilator, and thus we cannot assume that increasing the cerebral perfusion is uniquely responsible for the improved cognitive performance. Additionally, it has been reported that the enhancement of cognitive function in different intervention studies is related to an increase in blood BDNF levels, a protein associated with neuronal growth levels. In the brain, DBNF stimulates synaptic plasticity and neurogenesis and plays an important role in learning and memory functions. 

For obvious reasons, most of the literature available on human antiaging research, including polyphenol interventions, refer the effect in population groups of elders or, in most of them, groups suffering from chronic diseases. Therefore, less is known about the effect of polyphenol interventions on cognitive processes in healthy young humans and children but some of them, especially those related to cocoa and its derivatives, indicate improved brain function following acute and/or chronic ingestion of cocoa flavanols.

Overall, most findings support the beneficial effect of cocoa flavanols on cognitive function and neuroplasticity in young adults, suggesting that the inclusion of cocoa powder or high-cocoa flavanols products may be a realistic and reasonable preventive approach on neurodegenerative diseases and cognitive decline. Furthermore, short and middle-term effects of daily cocoa intake may provide young adults with a better cognitive performance in verbal learning, memory, and attention favoring academic achievement. Nevertheless, the available evidence is very scarce and future studies are needed to increase the robustness of the results.

## Figures and Tables

**Table 1:** Studies carried out with cocoa flavanols (CF) in a population averaging under 25 years and with neurological or cognitive outcomes.

Reference	Participants (Years Old)	Type of Study	Flavanol Amount	Control Group	Time *	Outcomes	Results
[33]	16 (female only) 18–30	acute/chronic (5 days)	172 mg CF for 5 days	13 mg CF	90 min	blood oxygenation level-dependent by fMRI	blood oxygenation in active brain regions fMRI
	4 24–31	acute	516 mg CF	39 mg	before, 2, 4& 6 h		cerebral blood flow increased 60% at 2 h after ingestion
[39]	30 (17 female) 21.9 ± 0.61	acute	520 mg & 994 mg of CF	46 mg	90 min	cognitive demand battery	improvement in serial 3 subtraction for all time point at 520 mg but not 994 mg and the same for fatigue improvement.
[40]	30 (22 female) 18–25	acute	773 mg CF (222 mg theobromine y 35 mg caffeine)	Trace amounts	2 h	visual contrast sensitivity and memory tasks	visual contrast sensitivity and integration time threshold, no effect on memory tasks
[37]	18 (11 female) 10.55 ± 1.45	chronic (10.11 ± 3.4 days)	680 mg CF (30 g cocoa)	no flavanol for 15 days.	4 h	Inflammation markers and short memory tests	decrease plasma endothelin1 and inflammatory mediators. In total, 83% of children showed a marginally significant improvement in one or both short memory tasks
[38]	40 (27 female) 24.13 ± 4.47	acute/chronic (28 days)	250 mg CF (Tablet with 3 g cocoa)	placebo tablet	2 h	subtraction, rapid visual processing, and a mental fatigue scale	improved self-reported mental fatigue and performance on the Serial Sevens task
[35]	32 (16 female) 25.31 ± 3.60	acute	520 mg CF	88.5 mg CF	90 min	cognitive functions in sleep deprivation conditions	working memory accuracy is higher only in female after high flavanol chocolate consumption in sleep deprivation conditions than after low flavanol in the same conditions
[34]	10 (0 female) 22.6 ± 0.3	acute	563 mg CF	38 mg CF (Energy matched beverage)	70 min	executive function and memory function (Stroop task, face-name matching)	CF could enhance exercise-induced improvement in executive function, but not in memory function
[41]	20 (ND females) 23.2 ± 4.3	acute	530 mg CF	Capsules matched for theobromine and caffeine	2 h	cognition battery and hemodynamic changes and neuronal activity	CF intake improved neurovascular coupling, but did not affect neuronal activity and cognitive performance in both normoxia and hypoxia
[42]	48 (24 female) 22.15 ± 0.01	acute	747 mg and 37 4 mg CF	alkalinized cocoa	2 h	cognition and visual functions	CF does not influence temporal attention, but does decrease reaction time in visual search with medium effect size and without losing accuracy
[36]	20 (6 female) 20–31	chronic (30 days)	24 g/day dark chocolate	White chocolate	20 h	cognitive function (Stroop and digital cancellation test). Prefrontal CBF	Increased Plasma Nerve Growth Factor, enhancing cognitive function performance
[43]	98 (57 female) 20.7 ± 0.18	acute	35 g dark chocolate	White chocolate	2 h	verbal episodic memory	dark chocolate consumption can benefit verbal episodic memory

* Time for outcome assessment.
